# Obesity as a driver of international differences in COVID‐19 death rates

**DOI:** 10.1111/dom.14357

**Published:** 2021-03-15

**Authors:** Julian Gardiner, Jude Oben, Alastair Sutcliffe

**Affiliations:** ^1^ University College London Great Ormond Street Institute of Child Health London UK; ^2^ Department of Education University of Oxford Oxford UK; ^3^ University College London Institute for Liver and Digestive Health Royal Free Hospital London UK; ^4^ Department of Gastroenterology and Hepatology Guy's and St Thomas' Hospital London UK

**Keywords:** observational study, obesity therapy, type 2 diabetes

## Abstract

**Aim:**

To determine what proportion of the inter‐country variation in death rates can be explained in terms of obesity rates and other known risk factors for coronavirus disease 2019 (COVID‐19).

**Materials and Methods:**

COVID‐19 death rates from 30 industrialized countries were analysed using linear regression models. Covariates modelled population density, the age structure of the population, obesity, population health, per capita gross domestic product (GDP), ethnic diversity, national temperature and the delay in the government imposing virus control measures.

**Results:**

The multivariable regression model explained 63% of the inter‐country variation in COVID‐19 death rates. The initial model was optimized using stepwise selection. In descending order of absolute size of model coefficient, the covariates in the optimized model were the obesity rate, the hypertension rate, population density, life expectancy, the percentage of the population aged older than 65 years, the percentage of the population aged younger than 15 years, the diabetes rate, the delay in imposing national COVID‐19 control measures, per capita GDP and mean temperature (with a negative coefficient indicating an association between higher national temperatures and lower death rates).

**Conclusions:**

A large proportion of the inter‐country variation in COVID‐19 death rates can be explained by differences in obesity rates, population health, population densities, age demographics, delays in imposing national virus control measures, per capita GDP and climate. Some of the unexplained variation is probably attributable to inter‐country differences in the definition of a COVID‐19 death and in the completeness of the recording of COVID‐19 deaths.

## 1 INTRODUCTION

The pandemic arising from the rapid spread of coronavirus disease 2019 (COVID‐19) has instigated unparalleled public health emergency measures from countries around the world. The number of deaths per million population varies widely internationally, and the reasons for these variations are not fully understood. In this paper, COVID‐19 death statistics from 30 industrialized countries are analysed in terms of demographic and health‐related covariates with a view to identifying explanatory factors underlying the inter‐country variation in mortality rates.

Government responses to the pandemic have varied greatly. Some countries, such as Norway and Denmark, imposed restrictions on the movement and association of citizens soon after the first case was identified in each country.^1^ Sweden, on the other hand, has largely relied on voluntary public health measures to control the spread of infection.^2^

Higher mortality rates have been reported among older adults^3^ and those with certain pre‐existing health conditions, including obesity,^4^, ^5^, ^6^ diabetes^7^, ^8^ and hypertension.^9^ Studies have also highlighted smoking as a risk factor for COVID‐19 mortality.^3^ There is conflicting evidence around the significance of ethnicity as a risk factor for mortality, which may be confounded by socioeconomic and medical disadvantages experienced by ethnic minorities.^10^, ^11^ There is also evidence of an interaction between the effects of obesity and ethnic group.^12^

It is widely acknowledged that greater population density facilitates the spread of COVID‐19.^13^ Because of the prolonged incubation period shown among children, some have suggested that they facilitate viral spread, although this is contentious.^14^, ^15^ Another factor implicated in the spread of respiratory viruses, such as COVID‐19, is the climate: there is growing evidence that COVID‐19 spreads more readily in temperate latitudes compared with hotter climates.^16^

Informed by this evidence, model covariates were drawn from the domains of age distribution, obesity and health, population density, socioeconomic factors and climate.

In addition to these demographic and environmental factors, a covariate was included, which measured how quickly governments responded to the coronavirus pandemic via severe restrictions on person‐to‐person contacts.

## 2 MATERIALS AND METHODS

To be included in the study countries had to have a population of at least 2 million and a per capita gross domestic product (GDP) of at least $27,000 (US) per year. Countries for which data were considered to be unreliable for data transparency reasons were excluded, for example, China and Russia. The selection of countries was made prior to data collection and data analysis. The following 30 countries were included in the analysis: Australia, Austria, Belgium, Canada, the Czech Republic, Denmark, Finland, France, Germany, Greece, Hungary, Ireland, Italy, Japan, Lithuania, Malaysia, the Netherlands, New Zealand, Norway, Poland, Portugal, Slovakia, Slovenia, South Korea, Spain, Sweden, Switzerland, Turkey, the UK and the United States. These countries make up 15% of the world's population.

The date of the first COVID‐19 case in each country was retrieved from the World Health Organization (WHO) website.^17^ The number of COVID‐19 deaths in each country up to 26 July 2020 was found from the John Hopkins University coronavirus resource centre.^18^ National populations and life expectancy data were sourced from the United Nations.^19^, ^20^ Data were retrieved from the World Bank regarding the percentage of each population living in urban settlements,^21^ the percentages of the population aged younger than 15 years and older than 65 years,^22^, ^23^ as well as the percentage of the population aged 20–79 years with diabetes.^24^ Per capita GDP and adult obesity rates were sourced from the Central Intelligence Agency (CIA) World Factbook.^25^, ^26^ The percentage of the adult population (aged ≥18 years) with hypertension, defined as a systolic blood pressure of 140 mmHg or higher, or a diastolic blood pressure of 90 mmHg or higher, was recorded from WHO data.^27^ Smoking rates were retrieved from the World Population Review,^28^ and mean national temperatures for 1961 to 1990 were sourced from the Climactic Research Unit.^29^ Population densities from official national estimates were retrieved from Wikipedia,^30^ as were ethnic diversity index figures,^31^ which were derived from Fearon.^32^

A country was considered to have introduced national COVID‐19 control measures once compulsory restrictions were adopted that applied to the whole country and included at least two of the following: a limit on public gatherings to 30 people or fewer; the closure of public buildings, including non‐essential shops, restaurants, bars, theatres and cinemas; the closure of schools; a requirement for the public to stay at home, except for medical reasons, exercise, essential shopping or work that could not be carried out remotely; a requirement for the public to observe social distancing. Determining when countries met these criteria for national COVID‐19 control measures required the consultation of a large number of sources. Most of these are accessible from the Wikipedia pages on the COVID‐19 pandemic in specific countries.^33^ Full details of the sources consulted are provided in the supporting information (Appendix S1). The delay in introducing national COVID‐19 control measures was taken to be the time from when the first case was reported to when these conditions were first met. For two countries, Sweden and South Korea, these criteria for national COVID‐19 control measures were never met. The delay in introducing national control measures was therefore considered to extend to 26 July 2020, the date up to which COVID‐19 deaths were counted.

The ethnic diversity index is defined as the probability that two randomly chosen individuals in a population come from different ethnic groups.^32^ For an ethnically homogenous population the index is zero, while for a population with many ethnic groups it increases towards a value of one.

The recording period was calculated from the date of the first case until 26 July 2020. The number of COVID‐19 deaths was divided by the population and the recording period to give a death rate in units of deaths per million population per year. COVID‐19 death rates were positively skewed. To produce a more normally distributed outcome variable and improve model fit, the outcome variable used was the log death rate.

An initial multivariable linear regression model was fitted of the log COVID‐19 death rate in terms of the covariates: population, population density, life expectancy, the percentage of the population living in urban settlements, the percentage of the population aged younger than 15 years, the percentage of the population aged older than 65 years, the percentage of the adult population that is obese, the percentage of the adult population with diabetes, the percentage of the adult population with hypertension, the percentage of the population who smoke, per capita GDP, ethnic diversity index, mean temperature and the delay in introducing national COVID‐19 control measures.

An optimized model (Model 1) was derived using stepwise covariate selection. This was carried out by minimizing the Akaike information criterion (AIC),^34^ a log‐likelihood penalized by the number of model variables. Minimizing this criterion produces a model that seeks to explain the variation in the outcome while omitting superfluous covariates. This was carried out using the stepAIC procedure in the MASS library for R.^35^, ^36^

The covariate ‘delay in introducing national COVID‐19 control measures’ differs from the others in that it was the result of government decisions. A further model (Model 2) was fitted that omitted this covariate from Model 1.

Countries were ranked by the model residuals from Models 1 and 2. These show the variation in death rates, which is not explained by the covariates in a given model. These rankings were compared with a ranking of countries by raw death rates.

Analyses were carried out in R 3.6.3.^35^

## 3 RESULTS

The date of the first case, the date upon which national COVID‐19 control measures were introduced, the delay in introducing national control measures, and the COVID‐19 death rate per million population per year are shown in Table 1. Summary statistics for all variables are given in Table 2.

**TABLE 1:** Date of first case, date upon which national measures were introduced, national measures delay and death rate per million population per year (raw and logged)

Country	Date of first case	Date national measures were introduced	National measures delay (days)	Death rate per million population per year	Log death rate per million population per year
Australia	25 January 2020	30 March 2020	65	11.1	2.41
Austria	26 February 2020	16 March 2020	19	192.0	5.26
Belgium	4 February 2020	17 March 2020	42	1795.2	7.49
Canada	26 January 2020	22 March 2020	56	478.4	6.17
Czech Republic	1 March 2020	16 March 2020	15	84.8	4.44
Denmark	27 February 2020	13 March 2020	15	258.6	5.56
Finland	29 January 2020	16 March 2020	47	121.3	4.80
France	24 January 2020	17 March 2020	53	920.0	6.82
Germany	28 January 2020	22 March 2020	54	221.5	5.40
Greece	27 February 2020	13 March 2020	15	46.7	3.84
Hungary	5 March 2020	16 March 2020	11	157.2	5.06
Ireland	1 March 2020	27 March 2020	26	897.2	6.80
Italy	29 January 2020	9 March 2020	40	1182.5	7.08
Japan	14 January 2020	16 April 2020	93	14.8	2.69
Lithuania	28 February 2020	16 March 2020	17	71.1	4.26
Malaysia	25 January 2020	18 March 2020	53	7.7	2.04
Netherlands	28 February 2020	15 March 2020	16	882.9	6.78
New Zealand	28 February 2020	25 March 2020	26	11.3	2.42
Norway	26 February 2020	12 March 2020	15	114.7	4.74
Poland	4 March 2020	12 March 2020	8	110.8	4.71
Portugal	2 March 2020	20 March 2020	18	417.1	6.03
Slovakia	6 March 2020	16 March 2020	10	13.2	2.58
Slovenia	4 March 2020	16 March 2020	12	140.3	4.94
South Korea	19 January 2020	26 July 2020	189	11.2	2.42
Spain	2 February 2020	28 March 2020	55	1269.5	7.15
Sweden	31 January 2020	26 July 2020	177	1171.3	7.07
Switzerland	24 February 2020	16 March 2020	21	549.3	6.31
Turkey	11 March 2020	16 March 2020	5	177.8	5.18
UK	1 February 2020	23 March 2020	51	1402.5	7.25
United States	20 January 2020	7 April 2020	78	852.9	6.75

**TABLE 2:** Summary statistics for variables

Variable	Minimum	Median	Maximum	Mean	SD
Population	2,078,654	11,114,268	329,064,917	39,347,743	62,949,691
Life expectancy (years)	75.7	81.8	84.5	81.0	2.3
Mean temperature (°C)	−5.3	9.1	25.4	9.3	5.8
% urban population	54.0	80.0	98.0	77.1	11.2
% <15 years of age	12.9	15.7	25.0	16.5	3.1
% >65 years of age	6.3	18.7	27.0	17.9	4.0
% obese	4.3	22.2	36.2	22.6	6.6
% with diabetes	3.2	6.0	16.7	6.7	2.8
% with hypertension	11.0	19.7	30.5	20.6	5.2
% who smoke	13.2	23.3	42.6	24.4	6.4
Per capita GDP (US $)	27,000	43,500	73,200	43,620	12,387
Population density (per km^2^)	3.0	106.1	516.7	140.9	129.5
Diversity index	0.004	0.210	0.857	0.269	0.218
National measures delay (days)	5	26	189	43	44
Number of COVID‐19 deaths	22	1324	144,469	11,368	27,794
Recording period (days)	137	152	194	163	18
Deaths per million population	3.8	73.0	850.3	209.5	245.5
Deaths per million population per year	7.7	184.9	1795.2	452.8	510.8
Log deaths per million population per year	2.04	5.22	7.49	5.15	1.70

Abbreviation: GDP, gross domestic product.

The correlation between obesity rate and COVID‐19 death rate (deaths per million population per year) was 0.153 for the raw death rate and 0.297 for the logged death rate.

The initial multivariable model including all covariates had a coefficient of determination (multiple R^2^) of 0.629, which indicates that 63% of the inter‐country variation in COVID‐19 death rates can be explained by the 14 covariates included in the model. Standardized model coefficients with 95% confidence intervals and associated *p*‐values are given in Table 3.

**TABLE 3:** Multivariable model of COVID‐19 death rate results in terms of all model covariates

Covariate	Standardized beta	95% confidence interval	*p*‐value
% obese	0.935	(0.257 to 1.612)	.010
% >65 years of age	0.928	(−0.224 to 2.080)	.107
Population density (per km^2^)	0.806	(0.231 to 1.381)	.009
% <15 years of age	0.750	(−0.166 to 1.665)	.101
% with hypertension	0.402	(−0.541 to 1.345)	.378
Life expectancy (years)	0.392	(−0.415 to 1.199)	.317
% with diabetes	0.383	(−0.157 to 0.924)	.152
National measures delay (days)	0.375	(−0.174 to 0.925)	.166
% urban population	−0.350	(−1.049 to 0.349)	.303
Mean temperature (°C)	−0.305	(−0.776 to 0.167)	.189
Per capita GDP (US $)	0.276	(−0.211 to 0.764)	.246
% smoking	0.175	(−0.269 to 0.619)	.415
Population	−0.133	(−0.698 to 0.431)	.622
Diversity index	0.112	(−0.356 to 0.580)	.617

Abbreviation: GDP, gross domestic product. Beta is a standardized regression model coefficient. Rows are sorted by the absolute value of beta, in descending order. The multiple R^2^ value was 0.629.

The model optimized using stepwise selection (Model 1) included 10 of the 14 covariates. In descending order of absolute size of the standardized model coefficients, these were the percentage of the adult population that is obese, the percentage of the adult population with hypertension, population density, life expectancy, the percentage of the population aged older than 65 years, the percentage of the population aged younger than 15 years, the percentage of the adult population with diabetes, national COVID‐19 control measures delay, per capita GDP and mean temperature. The model coefficient for mean temperature was negative, indicating an association between higher mean temperatures and lower COVID‐19 death rates. The multiple R^2^ value for this model was 0.583, showing that these 10 covariates explain 58% of the inter‐country variation in COVID‐19 death rates. Further results for Model 1 are given in Table 4.

**TABLE 4:** COVID‐19 death rate results in terms of an optimized set of covariates selected using AIC (Model 1), and the same model without the national measures delay covariate (Model 2)

Covariate	Model 1			Model 2		
	Standardized beta	95% confidence interval	*p*‐value	Standardized beta	95% confidence interval	*p*‐value
% obese	0.901	(0.347 to 1.455)	.003	0.719	(0.246 to 1.191)	.005
% with hypertension	0.689	(0.017 to 1.361)	.045	0.448	(−0.103 to 0.999)	.105
Population density (per km^2^)	0.688	(0.218 to 1.158)	.006	0.613	(0.153 to 1.072)	.011
Life expectancy (years)	0.519	(−0.126 to 1.163)	.109	0.437	(−0.201 to 1.075)	.169
% >65 years of age	0.438	(−0.200 to 1.076)	.167	0.440	(−0.206 to 1.086)	.171
% <15 years of age	0.353	(−0.249 to 0.955)	.234	0.344	(−0.265 to 0.953)	.253
% with diabetes	0.283	(−0.151 to 0.716)	.188	0.243	(−0.190 to 0.677)	.256
National measures delay	0.280	(−0.179 to 0.739)	.216			
Per capita GDP (US $)	0.279	(−0.153 to 0.712)	.193	0.235	(−0.197 to 0.667)	.269
Mean temperature (°C)	−0.265	(−0.667 to 0.138)	.185	−0.281	(−0.688 to 0.125)	.165

Abbreviations: AIC, Akaike information criterion; GDP, gross domestic product. Beta is a standardized regression model coefficient. Rows are sorted by the absolute value of beta, in descending order (Model 1). The multiple R^2^ values were 0.583 (Model 1) and 0.547 (Model 2).

Model 2, which omitted the covariate ‘national COVID‐19 control measures delay’, had a multiple R^2^ value of 0.547. Model 2 explains 3.6% less of the variation in death rates than Model 1 does. Further results for Model 2 are given in Table 4.

The rankings of countries by (A) raw COVID‐19 death rates, (B) residuals from Model 1 and (C) residuals from Model 2, are given in Table 5. Rankings (B) and (C) indicate the relative COVID‐19 death rates across countries once the variables in the relevant model have been controlled for.

**TABLE 5:** Countries ranked by (A) observed time‐adjusted death rate, (B) residuals from Model 1, and (C) residuals from Model 2

Rank	(A) Ranked by raw data	(B) Rank controlling for model covariates from optimized model (Model 1)		(C) Rank controlling for covariates, excluding national measures delay (Model 2)	
1	Belgium	Spain	+2	Spain	0
2	UK	Italy	+2	Sweden	+9
3	Spain	Portugal	+9	Italy	−1
4	Italy	Belgium	−3	France	+2
5	Sweden	Ireland	+2	Portugal	−2
6	France	France	0	Ireland	−1
7	Ireland	Malaysia	+23	Belgium	−3
8	Netherlands	Austria	+7	UK	+1
9	United States	UK	−7	Malaysia	−2
10	Switzerland	Switzerland	0	United States	+7
11	Canada	Sweden	−6	Austria	−3
12	Portugal	Denmark	+1	Canada	+1
13	Denmark	Canada	−2	Lithuania	+2
14	Germany	Greece	+10	Switzerland	−4
15	Austria	Lithuania	+8	Hungary	+3
16	Turkey	Poland	+5	Poland	0
17	Hungary	United States	−8	Denmark	−5
18	Slovenia	Hungary	−1	South Korea	+4
19	Finland	Finland	0	Greece	−5
20	Norway	Turkey	−4	Turkey	0
21	Poland	Netherlands	−13	Finland	−2
22	Czech Republic	South Korea	+6	Slovenia	+1
23	Lithuania	Slovenia	−5	Netherlands	−2
24	Greece	Norway	−4	Slovakia	+1
25	Japan	Slovakia	+1	Norway	−1
26	Slovakia	Germany	−12	Germany	0
27	New Zealand	Japan	−2	Czech Republic	+1
28	South Korea	Czech Republic	−6	Australia	+1
29	Australia	Australia	0	Japan	−2
30	Malaysia	New Zealand	−3	New Zealand	0

*Note:* The number following the Model 1 rankings (B) gives the change in rank compared with the raw data rankings (A). The number following the Model 2 rankings (C) gives the change in rank compared with the Model 1 rankings (B).

Where a country is lower in ranking (B) than ranking (A), this indicates that the country's death rate has been elevated by comparatively unfavourable values for the covariates included in Model 1. For example, the UK is seven places lower in ranking (B) than in ranking (A). This can be attributed to characteristics of the UK, including a high obesity rate, a high population density, a high proportion of older people and a low mean temperature.

Where a country is higher in ranking (B) than ranking (A), this indicates that the country's death rate has been depressed by comparatively favourable values for the Model 1 covariates. For example, Malaysia is 23 places higher in ranking (B) than in ranking (A). This can be attributed to characteristics of Malaysia, including a low obesity rate, a comparatively low percentage of the population aged older than 65 years and a high temperature.

Where a country is lower in ranking (C) than in ranking (B), this indicates that a comparatively short delay in introducing national COVID‐19 control measures helped to reduce the death rate. For example, Denmark had a delay in introducing control measures of 15 days and is five places lower in ranking (C) than in ranking (B).

Where a country is higher in ranking (C) than in ranking (B), this indicates that a comparatively long delay in introducing national COVID‐19 control measures contributed to increasing the death rate. For example, Sweden, which never introduced national COVID‐19 control measures as defined in this analysis, is ranked nine places higher in ranking (C) than in ranking (B), and the United States, which had a comparatively long delay of 78 days in introducing COVID‐19 control measures, is ranked seven places higher in ranking (C) than in ranking (B).

## 4 DISCUSSION

In the final multivariable model, the factor most strongly associated with COVID‐19 death rate is the obesity rate, followed by the hypertension rate then population density. Factors related to the age structure of the population show somewhat weaker associations, with higher life expectancy and a higher percentage of the population aged older than 65 years being associated with higher death rates, as would be expected given the greater vulnerability of older people to COVID‐19. A higher proportion of children aged younger than 15 years in the population is also associated with a higher death rate. The role of children in spreading COVID‐19 remains unclear, with recent evidence suggesting that children play less of a role than adults in transmitting the virus.^37^ However, it is possible that children have an important role in spreading the virus as asymptomatic carriers. Another possible explanation for this finding is that the role of children in spreading the virus is mainly an indirect one through population mixing, with children promoting adult‐to‐adult contact via school drop‐offs and pick‐ups, as well as adult‐to‐elder contact in situations where grandparents provide childcare.

The factors more weakly associated with COVID‐19 death rates are the diabetes rate, the delay in the imposition of national control measures, per capita GDP and temperature, with higher national temperatures being associated with lower death rates. Of these factors, only the role of per capita GDP is unexpected. A possible explanation for the association between higher levels of GDP and higher COVID‐19 death rates may be that there is more intra‐ and international travel in wealthier populations.

The covariates analysed here explain around 63% of the international variation in COVID‐19 death rates, leaving around 37% to be explained by other factors.

Some of this remaining variation is probably the effect on death statistics of heterogeneity in the ways in which a death from COVID‐19 has been defined in different countries. For example, some countries have considered that any death where the patient tested positive for COVID‐19 during their final illness was a death from COVID‐19, while other countries have only counted deaths where COVID‐19 was the main cause of death.^38^ Where there is a requirement for a positive test result, COVID‐19 deaths that occur outside hospital may not be recorded as such. Analysis of excess deaths in 2020, compared with the mean death rates for previous years, suggests that the number of COVID‐19 deaths may be considerably higher than has been reported, and the degree of under‐reporting probably varies by country.^39^

Sweden and South Korea differ from all the other countries studied in that neither imposed national COVID‐19 control measures, according to the definition adopted in the current study. The analysis suggests that the decision not to ‘lockdown’ led to a higher death rate in these countries than would otherwise have occurred. The death rate in the United States also appears to have been higher because of a slow imposition of national virus control measures, with the delay between the first case of COVID‐19 and the imposition of national measures being 78 days.

Concern has been expressed in the UK that the national COVID‐19 death rate is high compared with that of most other countries. The ‘lockdown’ delay in the UK was close to the international average, and therefore this by itself does not explain the UK's comparatively high death rate. This analysis suggests that the UK's high death rate is partly explained by demographic and health factors, including a high obesity rate, high population density and high rates of hypertension.

One strength of this analysis is the inclusion of a wide range of covariates resulting in a model that accounts for 63% of the inter‐country variation in COVID‐19 death rates. The countries studied comprise 15% of the global population, and therefore this represents a large proportion of the world's wealthier nations.

However, the sample size of 30 countries is small for linear regression analysis. This makes the results of the regression models less robust than where a larger sample size is available. Another limitation is that data were analysed at country level while there is considerable regional heterogeneity in the pandemic within countries. Some of the unexplained inter‐country variation in death rates is probably a result of modelling the outcome and covariates at this comparatively high level.

In conclusion, a considerable proportion of the international variation in COVID‐19 death rates in wealthier countries can be explained by obesity rates, population density, age distribution, hypertension, diabetes rates and national temperature, all factors which are outside government control, at least in the short term.

However, how quickly governments imposed virus control measures was also an influential factor on death rates, and this should inform policy in the case of future viral epidemics. There is also a case for strengthening policies aimed at tackling obesity, diabetes and hypertension, because an additional benefit of such public health measures may be that populations have greater resistance to future respiratory virus epidemics. The relationships between obesity, diabetes and COVID‐19 are not yet fully understood.^40^ Further studies are needed to clarify how obesity, diabetes and hypertension influence death rates from respiratory epidemics such as COVID‐19.

## CONFLICT OF INTEREST

The authors have no conflicts of interest to declare.

## AUTHOR CONTRIBUTIONS

All authors contributed to the design of the study. Data analyses were conducted primarily by Dr Gardiner. All authors verify the authenticity of the report.

### PEER REVIEW

The peer review history for this article is available at https://publons.com/publon/10.1111/dom.14357.

## Supporting information


**Appendix S1.** Supporting Information.

## Data Availability

The complete analysis of data has been made available in the online Supporting Information.

## References

[1] Olagnier D , Mogensen TH . The Covid‐19 pandemic in Denmark: big lessons from a small country. Cytokine Growth Factor Rev. 2020;53:10‐12. 32405247 10.1016/j.cytogfr.2020.05.005 PMC7217796

[2] Kamerlin SCL , Kasson PM . Managing COVID‐19 spread with voluntary public‐health measures: Sweden as a case study for pandemic control. Clin Infect Dis. 2020;71:3174‐3181. 32609825 10.1093/cid/ciaa864 PMC7337695

[3] Zheng Z , Peng F , Xu B , et al. Risk factors of critical & mortal COVID‐19 cases: a systematic literature review and meta‐analysis. J Infect. 2020;81:e16‐e25. 10.1016/j.jinf.2020.04.021 PMC7177098 32335169

[4] Dietz W , Santos‐Burgoa C . Obesity and its implications for COVID‐19 mortality. Obesity. 2020;28:1005. 32237206 10.1002/oby.22818

[5] Peters SAE , MacMahon S , Woodward M . Obesity as a risk factor for COVID‐19 mortality in women and men in the UK biobank: comparisons with influenza/pneumonia and coronary heart disease. Diabetes Obes Metab. 2021;23:258‐262. 32969132 10.1111/dom.14199 PMC7536945

[6] Luo XM , Jiaerken YM , Shen ZM , et al. Obese COVID‐19 patients show more severe pneumonia lesions on CT chest imaging. Diabetes Obes Metab. 2021;23:290‐293. 32945051 10.1111/dom.14194 PMC7537264

[7] Zhang Y , Li H , Zhang J , et al. The clinical characteristics and outcomes of patients with diabetes and secondary hyperglycaemia with coronavirus disease 2019: a single‐centre, retrospective, observational study in Wuhan. Diabetes Obes Metab. 2020;22:1443‐1454. 32406594 10.1111/dom.14086 PMC7273002

[8] Wu J , Zhang J , Sun X , et al. Influence of diabetes mellitus on the severity and fatality of SARS‐CoV‐2 (COVID‐19) infection. Diabetes Obes Metab. 2020;22:1907‐1914. 32496012 10.1111/dom.14105 PMC7300679

[9] Fang L , Karakiulakis G , Roth M . Are patients with hypertension and diabetes mellitus at increased risk for COVID‐19 infection? Lancet Respir Med. 2020;8:e21. 32171062 10.1016/S2213-2600(20)30116-8 PMC7118626

[10] Price‐Haywood EG , Burton J , Fort D , Seoane L . Hospitalization and mortality among black patients and white patients with Covid‐19. N Engl J Med. 2020;382:2534‐2543. 32459916 10.1056/NEJMsa2011686 PMC7269015

[11] Evans MK . Covid's color line ‐ infectious disease, inequity, and racial justice. N Engl J Med. 2020;383:408‐410. 32726526 10.1056/NEJMp2019445

[12] Razieh C , Zaccardi F , Davies MJ , Khunti K , Yates T . Body mass index and the risk of COVID‐19 across ethnic groups: analysis of UK Biobank. Diabetes Obes Metab. 2020;22:1953‐1954. 32602268 10.1111/dom.14125 PMC7362044

[13] Rocklov J , Sjodin H . High population densities catalyse the spread of COVID‐19. J Travel Med. 2020;27. 10.1093/jtm/taaa038. PMC7184409 32227186

[14] She J , Liu L , Liu W . COVID‐19 epidemic: disease characteristics in children. J Med Virol. 2020;92:747‐754. 32232980 10.1002/jmv.25807 PMC7228385

[15] Snelson E , Roland D , Munro APS . Throat and ear infections in children: URTI in the time of COVID‐19. Arch Dis Child Educ Pract Ed. 2020;1‐3. 10.1136/archdischild-2020-318854. 32591362

[16] O'Reilly KM , Auzenbergs M , Jafari Y , Liu Y , Flasche S , Lowe R . Effective transmission across the globe: the role of climate in COVID‐19 mitigation strategies. Lancet Planet Health. 2020;4:e172. 32389182 10.1016/S2542-5196(20)30106-6 PMC7202845

[17] The World Health Organisation . WHO Coronavirus Disease (COVID‐19) Dashboard. 2020. https://covid19.who.int/?gclid=CjwKCAjwydP5BRBREiwA‐qrCGkBRL8EBFdFLVJ2WYqhvkUEqknMXbchu7Yf1ZBdMYO8cZX0y3ePcTxoCc7gQAvD_BwE. Accessed August 13, 2020.

[18] The Johns Hopkins Coronavirus Resource Center. COVID‐19 Dashboard. 2020. https://coronavirus.jhu.edu/map.html. Accessed July 26, 2020.

[19] United Nations Data. Total population, both sexes combined 2020. https://data.un.org/Data.aspx?d=PopDiv&f=variableID%3a12%3btimeID%3a83%2c84%3bvarID%3a2&c=2,4,6,7&s=_crEngNameOrderBy:asc,_timeEngNameOrderBy:desc,_varEngNameOrderBy:asc&v=1. Accessed July 24, 2020.

[20] United Nations Human Development Reports. 2019 Human Development Index Ranking. 2020. http://hdr.undp.org/en/content/2019-human-development-index-ranking. Accessed July 24, 2020.

[21] The World Bank. Urban Population (% of total population). 2020. https://data.worldbank.org/indicator/SP.URB.TOTL.IN.ZS. Accessed July 24, 2020.

[22] The World Bank. Population ages 0‐14 (% of total population). 2020. https://data.worldbank.org/indicator/SP.POP.0014.TO.ZS. Accessed July 24, 2020.

[23] The World Bank. Population ages 65 and above (% of total population). 2020. https://data.worldbank.org/indicator/SP.POP.65UP.TO.ZS?name_desc=false&view=chart. Accessed July 24, 2020.

[24] Diabetes Prevalence (% of adult population ages 20 to 79) ; 2020. https://data.worldbank.org/indicator/SH.STA.DIAB.ZS. Accessed July 27, 2020.

[25] The World Factbook: GDP per capita ; 2020. https://www.cia.gov/library/publications/the-world-factbook/fields/211rank.html. Accessed July 24, 2020.

[26] The World Factbook: Obesity ; 2020. https://www.cia.gov/library/publications/the-world-factbook/rankorder/2228rank.html. Accessed July 24, 2020.

[27] Raised blood pressure (SBP>=140 OR DBP>=90) (age‐standardized estimate) ; 2020. https://www.who.int/data/gho/data/indicators/indicator‐details/GHO/raised‐blood‐pressure‐(sbp‐=140‐or‐dbp‐=90)‐(age‐standardized‐estimate). Accessed July 27, 2020.

[28] Smoking Rates By Country 2020 ; 2020. https://worldpopulationreview.com/country-rankings/smoking-rates-by-country. Accessed August 11, 2020.

[29] Average yearly temperature (1961‐1990, Celsius) ‐ by country ; 2020. https://web.archive.org/web/20150905135247/http:/lebanese‐economy‐forum.com/wdi‐gdf‐advanced‐data‐display/show/EN‐CLC‐AVRT‐C/. Accessed July 24, 2020.

[30] List of countries and dependencies by population density ; 2020. https://en.wikipedia.org/wiki/List_of_countries_and_dependencies_by_population_density. Accessed August 11, 2020.

[31] List of countries ranked by ethnic and cultural diversity level ; 2020. https://en.wikipedia.org/wiki/List_of_countries_ranked_by_ethnic_and_cultural_diversity_level. Accessed August 11, 2020.

[32] Fearon JD . Ethnic and cultural diversity by country. J Econ Growth. 2003;8:195‐222.

[33] Wikipedia. COVID‐19 pandemic by country and territory. 2020. https://en.wikipedia.org/wiki/COVID-19_pandemic_by_country_and_territory. Accessed August 11, 2020.

[34] Akaike H . A new look at the statistical model identification. IEEE Trans Autom Control. 1974;19:716‐723.

[35] R Core Team . R: A Language and Environment for Statistical Computing. Vienna, Austria; R Foundation for Statistical Computing; 2020.

[36] Ripley B , Venables B . MASS: Support Functions and Datasets for Venables and Ripley's MASS. 2020. https://cran.r-project.org/web/packages/MASS/index.html. Accessed August 1, 2020.

[37] Viner RM , Mytton OT , Bonell C , et al. Susceptibility to SARS‐CoV‐2 infection among children and adolescents compared with adults: a systematic review and meta‐analysis. JAMA Pediatr. 2021;175:143‐156. 32975552 10.1001/jamapediatrics.2020.4573 PMC7519436

[38] COVID‐19 Health System Response Monitor. How comparable is covid‐19 mortality across countries? 2020. https://analysis.covid19healthsystem.org/index.php/2020/06/04/how-comparable-is-covid-19-mortality-across-countries/. Accessed September 15, 2020.

[39] Burn‐Murdoch, J., Romei, V., Giles C. Global coronavirus death toll could be 60% higher than reported. Financial Times, 2020. https://www.ft.com/content/6bd88b7d-3386-4543-b2e9-0d5c6fac846c. Accessed September 15, 2020.

[40] Vas P , Hopkins D , Feher M , Rubino F , Whyte MB . Diabetes, obesity and COVID‐19: a complex interplay. Diabetes Obes Metab. 2020;22:1892‐1896. 32627299 10.1111/dom.14134 PMC7362013

