# Supplementary material for: Obesity as a driver of international differences in COVID‐19 death rates
Source: Diabetes Obes Metab. 2021 Mar 15;23(7):1463–70. doi: 10.1111/dom.14357 (PMC8013490; doi:10.1111/dom.14357)
Supplement: Supplementary file 1 — Appendix S1. Supporting Information. [file DOM-23-1463-s001.docx]

**Supporting Information**

**Contents**

[Introduction 2](#_Toc61855724)

[Materials and methods 3](#_Toc61855725)

[Results 4](#_Toc61855726)

[Discussion 6](#_Toc61855727)

[References 8](#_Toc61855728)

[Tables 11](#_Toc61855729)

[Tables of complete analysis data 16](#_Toc61855730)

[Tables of countries ranked by each variable 22](#_Toc61855731)

[Comparing the outcome variable with the model residuals 34](#_Toc61855732)

[Data sources from which national measures dates were derived 36](#_Toc61855733)

# Tables of complete analysis data

The raw data used in the analysis are shown in Tables 6 to 10.

- Population, population density, life expectancy, per capita GDP and Diversity index. (Table 6)
- Health variables. (Table 7)
- Temperature, % urban population, % under age 15 and % over age 65. (Table 8)
- COVID-19 deaths and related variables. (Table 9)
- Date of first case and date national measures were introduced. (Table 10).

Table 6: Raw data: population, population density, life expectancy, per capita GDP and Diversity index.

| **Country** | **Population** | **Population density (per km^2^)** | **Life Expectancy (years)** | **Per capita GDP (US $)** | **Diversity index** |
| --- | --- | --- | --- | --- | --- |
| Australia | 25,203,198 | 3.00 | 83.3 | 50,400 | 0.857 |
| Austria | 8,955,102 | 106.14 | 81.4 | 50,000 | 0.126 |
| Belgium | 11,539,328 | 375.52 | 81.5 | 46,600 | 0.567 |
| Canada | 37,411,047 | 4.00 | 82.3 | 48,400 | 0.596 |
| Czech Republic | 10,689,209 | 135.43 | 79.2 | 35,500 | 0.322 |
| Denmark | 5,771,876 | 134.91 | 80.8 | 50,100 | 0.128 |
| Finland | 5,532,156 | 16.33 | 81.7 | 44,500 | 0.132 |
| France | 65,129,728 | 123.28 | 82.5 | 44,100 | 0.272 |
| Germany | 83,517,045 | 232.80 | 81.2 | 50,800 | 0.095 |
| Greece | 10,473,455 | 81.27 | 82.1 | 27,800 | 0.059 |
| Hungary | 9,684,679 | 104.96 | 76.7 | 29,600 | 0.186 |
| Ireland | 4,882,495 | 70.03 | 82.1 | 73,200 | 0.171 |
| Italy | 60,550,075 | 199.97 | 83.6 | 38,200 | 0.040 |
| Japan | 126,860,301 | 333.38 | 84.5 | 42,900 | 0.012 |
| Lithuania | 2,759,627 | 42.78 | 75.7 | 32,400 | 0.338 |
| Malaysia | 31,949,777 | 99.00 | 76.1 | 29,100 | 0.596 |
| Netherlands | 17,097,130 | 421.00 | 82.1 | 53,900 | 0.077 |
| New Zealand | 4,783,063 | 19.00 | 82.1 | 39,000 | 0.363 |
| Norway | 5,378,857 | 16.58 | 82.3 | 72,100 | 0.098 |
| Poland | 37,887,768 | 122.76 | 78.5 | 29,600 | 0.047 |
| Portugal | 10,226,187 | 111.59 | 81.9 | 30,500 | 0.040 |
| Slovakia | 5,457,013 | 111.15 | 77.4 | 33,100 | 0.332 |
| Slovenia | 2,078,654 | 102.81 | 81.2 | 34,500 | 0.231 |
| South Korea | 51,225,308 | 516.72 | 82.8 | 39,500 | 0.004 |
| Spain | 46,736,776 | 92.76 | 83.4 | 38,400 | 0.502 |
| Sweden | 10,036,379 | 22.97 | 82.7 | 51,200 | 0.189 |
| Switzerland | 8,591,365 | 207.98 | 83.4 | 62,100 | 0.575 |
| Turkey | 83,429,615 | 106.12 | 78.3 | 27,000 | 0.299 |
| United Kingdom | 67,530,172 | 279.95 | 81.2 | 44,300 | 0.324 |
| United States | 329,064,917 | 34.00 | 78.9 | 59,800 | 0.491 |

Table 7: Raw data: health variables.

| **Country** | **% obese** | **% with diabetes** | **% with hypertension** | **% smoking** |
| --- | --- | --- | --- | --- |
| Australia | 29.0 | 5.6 | 15.2 | 14.90 |
| Austria | 20.1 | 6.6 | 21.0 | 35.15 |
| Belgium | 22.1 | 4.6 | 17.5 | 23.25 |
| Canada | 29.4 | 7.6 | 13.2 | 14.95 |
| Czech Republic | 26.0 | 7.0 | 27.9 | 33.20 |
| Denmark | 19.7 | 8.3 | 20.6 | 17.00 |
| Finland | 22.2 | 5.6 | 19.4 | 20.85 |
| France | 21.6 | 4.8 | 22.0 | 27.70 |
| Germany | 22.3 | 10.4 | 19.9 | 30.35 |
| Greece | 24.9 | 4.7 | 19.1 | 42.65 |
| Hungary | 26.4 | 6.9 | 30.0 | 28.40 |
| Ireland | 25.3 | 3.2 | 19.7 | 22.15 |
| Italy | 19.9 | 5.0 | 21.2 | 24.00 |
| Japan | 4.3 | 5.6 | 17.6 | 22.15 |
| Lithuania | 26.3 | 3.8 | 29.3 | 30.15 |
| Malaysia | 15.6 | 16.7 | 22.9 | 22.20 |
| Netherlands | 20.4 | 5.4 | 18.7 | 25.05 |
| New Zealand | 30.8 | 6.2 | 16.2 | 13.20 |
| Norway | 23.1 | 5.3 | 19.7 | 22.25 |
| Poland | 23.1 | 6.1 | 28.7 | 28.05 |
| Portugal | 20.8 | 9.8 | 24.4 | 22.60 |
| Slovakia | 20.5 | 6.5 | 28.5 | 28.65 |
| Slovenia | 20.2 | 5.9 | 30.5 | 20.20 |
| South Korea | 4.7 | 6.9 | 11.0 | 27.00 |
| Spain | 23.8 | 6.9 | 19.2 | 29.20 |
| Sweden | 20.6 | 4.8 | 19.3 | 20.60 |
| Switzerland | 19.5 | 5.7 | 18.0 | 23.30 |
| Turkey | 32.1 | 11.1 | 20.3 | 25.95 |
| United Kingdom | 27.8 | 3.9 | 15.2 | 19.15 |
| United States | 36.2 | 10.8 | 12.9 | 17.25 |

Table 8: Raw data: temperature, % urban population, % under age 15 and % over age 65.

| **Country** | **Mean Temperature (℃)** | **% urban population** | **% under age 15** | **% over age 65** |
| --- | --- | --- | --- | --- |
| Australia | 21.65 | 86 | 19.0 | 15.5 |
| Austria | 6.35 | 59 | 14.1 | 19.2 |
| Belgium | 9.55 | 98 | 17.1 | 18.6 |
| Canada | -5.35 | 81 | 16.0 | 17.0 |
| Czech Republic | 7.55 | 74 | 15.4 | 19.0 |
| Denmark | 7.50 | 88 | 16.5 | 19.7 |
| Finland | 1.70 | 85 | 16.4 | 21.2 |
| France | 10.70 | 81 | 18.1 | 19.7 |
| Germany | 8.50 | 77 | 13.1 | 21.5 |
| Greece | 15.40 | 79 | 14.2 | 20.4 |
| Hungary | 9.75 | 72 | 14.3 | 18.6 |
| Ireland | 9.30 | 63 | 21.6 | 13.9 |
| Italy | 13.45 | 71 | 13.5 | 23.0 |
| Japan | 11.15 | 92 | 12.9 | 27.0 |
| Lithuania | 6.20 | 68 | 14.8 | 19.0 |
| Malaysia | 25.40 | 77 | 24.3 | 6.3 |
| Netherlands | 9.25 | 92 | 16.4 | 18.8 |
| New Zealand | 10.55 | 87 | 19.8 | 15.3 |
| Norway | 1.50 | 83 | 17.8 | 16.8 |
| Poland | 7.85 | 60 | 14.8 | 16.8 |
| Portugal | 15.15 | 66 | 13.6 | 21.5 |
| Slovakia | 6.80 | 54 | 15.4 | 15.1 |
| Slovenia | 8.90 | 55 | 15.0 | 19.1 |
| South Korea | 11.50 | 81 | 13.5 | 13.9 |
| Spain | 13.30 | 81 | 14.7 | 19.4 |
| Sweden | 2.10 | 88 | 17.5 | 19.9 |
| Switzerland | 5.50 | 74 | 14.9 | 18.4 |
| Turkey | 11.10 | 76 | 25.0 | 8.2 |
| United Kingdom | 8.45 | 84 | 17.7 | 18.5 |
| United States | 8.55 | 82 | 18.9 | 15.4 |

Table 9: Raw data: COVID-19 deaths and related variables.

| **Country** | **No. of COVID deaths** | **Recording period (days)** | **Deaths per million population** | **Deaths per million population per year** | **Log deaths per million population per year** |
| --- | --- | --- | --- | --- | --- |
| Australia | 140 | 183 | 5.6 | 11.1 | 2.41 |
| Austria | 711 | 151 | 79.4 | 192.0 | 5.26 |
| Belgium | 9812 | 173 | 850.3 | 1795.2 | 7.49 |
| Canada | 8919 | 182 | 238.4 | 478.4 | 6.17 |
| Czech Republic | 365 | 147 | 34.1 | 84.8 | 4.44 |
| Denmark | 613 | 150 | 106.2 | 258.6 | 5.56 |
| Finland | 329 | 179 | 59.5 | 121.3 | 4.80 |
| France | 30185 | 184 | 463.5 | 920.0 | 6.82 |
| Germany | 9117 | 180 | 109.2 | 221.5 | 5.40 |
| Greece | 201 | 150 | 19.2 | 46.7 | 3.84 |
| Hungary | 596 | 143 | 61.5 | 157.2 | 5.06 |
| Ireland | 1763 | 147 | 361.1 | 897.2 | 6.80 |
| Italy | 35092 | 179 | 579.6 | 1182.5 | 7.08 |
| Japan | 994 | 194 | 7.8 | 14.8 | 2.69 |
| Lithuania | 80 | 149 | 29.0 | 71.1 | 4.26 |
| Malaysia | 123 | 183 | 3.8 | 7.7 | 2.04 |
| Netherlands | 6158 | 149 | 360.2 | 882.9 | 6.78 |
| New Zealand | 22 | 149 | 4.6 | 11.3 | 2.42 |
| Norway | 255 | 151 | 47.4 | 114.7 | 4.74 |
| Poland | 1655 | 144 | 43.7 | 110.8 | 4.71 |
| Portugal | 1705 | 146 | 166.7 | 417.1 | 6.03 |
| Slovakia | 28 | 142 | 5.1 | 13.2 | 2.58 |
| Slovenia | 115 | 144 | 55.3 | 140.3 | 4.94 |
| South Korea | 298 | 189 | 5.8 | 11.2 | 2.42 |
| Spain | 28429 | 175 | 608.3 | 1269.5 | 7.15 |
| Sweden | 5697 | 177 | 567.6 | 1171.3 | 7.07 |
| Switzerland | 1977 | 153 | 230.1 | 549.3 | 6.31 |
| Turkey | 5563 | 137 | 66.7 | 177.8 | 5.18 |
| United Kingdom | 45639 | 176 | 675.8 | 1402.5 | 7.25 |
| United States | 144469 | 188 | 439.0 | 852.9 | 6.75 |

Table 10: Raw data: Date of first case and date national measures were introduced.

| **Country** | **Date of First Case** | **National measures date** | **National measures delay (days)** |
| --- | --- | --- | --- |
| Australia | 25 January 2020 | 30 March 2020 | 65 |
| Austria | 26 February 2020 | 16 March 2020 | 19 |
| Belgium | 04 February 2020 | 17 March 2020 | 42 |
| Canada | 26 January 2020 | 22 March 2020 | 56 |
| Czech Republic | 01 March 2020 | 16 March 2020 | 15 |
| Denmark | 27 February 2020 | 13 March 2020 | 15 |
| Finland | 29 January 2020 | 16 March 2020 | 47 |
| France | 24 January 2020 | 17 March 2020 | 53 |
| Germany | 28 January 2020 | 22 March 2020 | 54 |
| Greece | 27 February 2020 | 13 March 2020 | 15 |
| Hungary | 05 March 2020 | 16 March 2020 | 11 |
| Ireland | 01 March 2020 | 27 March 2020 | 26 |
| Italy | 29 January 2020 | 09 March 2020 | 40 |
| Japan | 14 January 2020 | 16 April 2020 | 93 |
| Lithuania | 28 February 2020 | 16 March 2020 | 17 |
| Malaysia | 25 January 2020 | 18 March 2020 | 53 |
| Netherlands | 28 February 2020 | 15 March 2020 | 16 |
| New Zealand | 28 February 2020 | 25 March 2020 | 26 |
| Norway | 26 February 2020 | 12 March 2020 | 15 |
| Poland | 04 March 2020 | 12 March 2020 | 8 |
| Portugal | 02 March 2020 | 20 March 2020 | 18 |
| Slovakia | 06 March 2020 | 16 March 2020 | 10 |
| Slovenia | 04 March 2020 | 16 March 2020 | 12 |
| South Korea | 19 January 2020 | 26 July 2020 | 189 |
| Spain | 02 February 2020 | 28 March 2020 | 55 |
| Sweden | 31 January 2020 | 26 July 2020 | 177 |
| Switzerland | 24 February 2020 | 16 March 2020 | 21 |
| Turkey | 11 March 2020 | 16 March 2020 | 5 |
| United Kingdom | 01 February 2020 | 23 March 2020 | 51 |
| United States | 20 January 2020 | 07 April 2020 | 78 |

# Tables of countries ranked by each variable

Countries are shown ranked by each variable in Tables 11 to 21.

- Countries ranked by population and per capita GDP. (Table 11)
- Countries ranked by population density and % urban population. (Table 12)
- Countries ranked by % under age 15 and % over age 65. (Table 13)
- Countries ranked by life expectancy and Diversity index. (Table 14)
- Countries ranked by mean temperature. (Table 15)
- Countries ranked by % of adult population which is obese and % of adult population with diabetes. (Table 16)
- Countries ranked by % of adult population with hypertension and % of adult population which smokes. (Table 17)
- Countries ranked by numbers of COVID-19 deaths and period from first case to date when death totals were retrieved. (Table 18)
- Countries ranked by death rate (deaths per million population) and time-adjusted death rate (deaths per million population per year) / log time-adjusted death rate. (Table 19)
- Countries ranked by date of first COVID-19 case. (Table 20)
- Countries ranked by date national measures were introduced and delay between first case and the introduction of national measures. (Table 21)

Table 11: Countries ranked by population and per capita GDP.

| **Country** | **Population** | **Country** | **Per capita GDP (US $)** |
| --- | --- | --- | --- |
| United States | 329,064,917 | Ireland | 73,200 |
| Japan | 126,860,301 | Norway | 72,100 |
| Germany | 83,517,045 | Switzerland | 62,100 |
| Turkey | 83,429,615 | United States | 59,800 |
| United Kingdom | 67,530,172 | Netherlands | 53,900 |
| France | 65,129,728 | Sweden | 51,200 |
| Italy | 60,550,075 | Germany | 50,800 |
| South Korea | 51,225,308 | Australia | 50,400 |
| Spain | 46,736,776 | Denmark | 50,100 |
| Poland | 37,887,768 | Austria | 50,000 |
| Canada | 37,411,047 | Canada | 48,400 |
| Malaysia | 31,949,777 | Belgium | 46,600 |
| Australia | 25,203,198 | Finland | 44,500 |
| Netherlands | 17,097,130 | United Kingdom | 44,300 |
| Belgium | 11,539,328 | France | 44,100 |
| Czech Republic | 10,689,209 | Japan | 42,900 |
| Greece | 10,473,455 | South Korea | 39,500 |
| Portugal | 10,226,187 | New Zealand | 39,000 |
| Sweden | 10,036,379 | Spain | 38,400 |
| Hungary | 9,684,679 | Italy | 38,200 |
| Austria | 8,955,102 | Czech Republic | 35,500 |
| Switzerland | 8,591,365 | Slovenia | 34,500 |
| Denmark | 5,771,876 | Slovakia | 33,100 |
| Finland | 5,532,156 | Lithuania | 32,400 |
| Slovakia | 5,457,013 | Portugal | 30,500 |
| Norway | 5,378,857 | Hungary | 29,600 |
| Ireland | 4,882,495 | Poland | 29,600 |
| New Zealand | 4,783,063 | Malaysia | 29,100 |
| Lithuania | 2,759,627 | Greece | 27,800 |
| Slovenia | 2,078,654 | Turkey | 27,000 |

Table 12: Countries ranked by population density and % urban population.

| **Country** | **Population density (per km^2^)** | **Country** | **% urban population** |
| --- | --- | --- | --- |
| South Korea | 516.72 | Belgium | 98 |
| Netherlands | 421.00 | Japan | 92 |
| Belgium | 375.52 | Netherlands | 92 |
| Japan | 333.38 | Denmark | 88 |
| United Kingdom | 279.95 | Sweden | 88 |
| Germany | 232.80 | New Zealand | 87 |
| Switzerland | 207.98 | Australia | 86 |
| Italy | 199.97 | Finland | 85 |
| Czech Republic | 135.43 | United Kingdom | 84 |
| Denmark | 134.91 | Norway | 83 |
| France | 123.28 | United States | 82 |
| Poland | 122.76 | Canada | 81 |
| Portugal | 111.59 | France | 81 |
| Slovakia | 111.15 | South Korea | 81 |
| Austria | 106.14 | Spain | 81 |
| Turkey | 106.12 | Greece | 79 |
| Hungary | 104.96 | Germany | 77 |
| Slovenia | 102.81 | Malaysia | 77 |
| Malaysia | 99.00 | Turkey | 76 |
| Spain | 92.76 | Czech Republic | 74 |
| Greece | 81.27 | Switzerland | 74 |
| Ireland | 70.03 | Hungary | 72 |
| Lithuania | 42.78 | Italy | 71 |
| United States | 34.00 | Lithuania | 68 |
| Sweden | 22.97 | Portugal | 66 |
| New Zealand | 19.00 | Ireland | 63 |
| Norway | 16.58 | Poland | 60 |
| Finland | 16.33 | Austria | 59 |
| Canada | 4.00 | Slovenia | 55 |
| Australia | 3.00 | Slovakia | 54 |

Table 13: Countries ranked by % under age 15 and % over age 65.

| **Country** | **% under age 15** | **Country** | **% over age 65** |
| --- | --- | --- | --- |
| Turkey | 25.0 | Japan | 27.0 |
| Malaysia | 24.3 | Italy | 23.0 |
| Ireland | 21.6 | Germany | 21.5 |
| New Zealand | 19.8 | Portugal | 21.5 |
| Australia | 19.0 | Finland | 21.2 |
| United States | 18.9 | Greece | 20.4 |
| France | 18.1 | Sweden | 19.9 |
| Norway | 17.8 | Denmark | 19.7 |
| United Kingdom | 17.7 | France | 19.7 |
| Sweden | 17.5 | Spain | 19.4 |
| Belgium | 17.1 | Austria | 19.2 |
| Denmark | 16.5 | Slovenia | 19.1 |
| Finland | 16.4 | Czech Republic | 19.0 |
| Netherlands | 16.4 | Lithuania | 19.0 |
| Canada | 16.0 | Netherlands | 18.8 |
| Czech Republic | 15.4 | Belgium | 18.6 |
| Slovakia | 15.4 | Hungary | 18.6 |
| Slovenia | 15.0 | United Kingdom | 18.5 |
| Switzerland | 14.9 | Switzerland | 18.4 |
| Lithuania | 14.8 | Canada | 17.0 |
| Poland | 14.8 | Norway | 16.8 |
| Spain | 14.7 | Poland | 16.8 |
| Hungary | 14.3 | Australia | 15.5 |
| Greece | 14.2 | United States | 15.4 |
| Austria | 14.1 | New Zealand | 15.3 |
| Portugal | 13.6 | Slovakia | 15.1 |
| Italy | 13.5 | Ireland | 13.9 |
| South Korea | 13.5 | South Korea | 13.9 |
| Germany | 13.1 | Turkey | 8.2 |
| Japan | 12.9 | Malaysia | 6.3 |

Table 14: Countries ranked by life expectancy and Diversity index.

| **Country** | **Life Expectancy (years)** | **Country** | **Diversity index** |
| --- | --- | --- | --- |
| Japan | 84.5 | Australia | 0.857 |
| Italy | 83.6 | Canada | 0.596 |
| Spain | 83.4 | Malaysia | 0.596 |
| Switzerland | 83.4 | Switzerland | 0.575 |
| Australia | 83.3 | Belgium | 0.567 |
| South Korea | 82.8 | Spain | 0.502 |
| Sweden | 82.7 | United States | 0.491 |
| France | 82.5 | New Zealand | 0.363 |
| Canada | 82.3 | Lithuania | 0.338 |
| Norway | 82.3 | Slovakia | 0.332 |
| Greece | 82.1 | United Kingdom | 0.324 |
| Ireland | 82.1 | Czech Republic | 0.322 |
| Netherlands | 82.1 | Turkey | 0.299 |
| New Zealand | 82.1 | France | 0.272 |
| Portugal | 81.9 | Slovenia | 0.231 |
| Finland | 81.7 | Sweden | 0.189 |
| Belgium | 81.5 | Hungary | 0.186 |
| Austria | 81.4 | Ireland | 0.171 |
| Germany | 81.2 | Finland | 0.132 |
| Slovenia | 81.2 | Denmark | 0.128 |
| United Kingdom | 81.2 | Austria | 0.126 |
| Denmark | 80.8 | Norway | 0.098 |
| Czech Republic | 79.2 | Germany | 0.095 |
| United States | 78.9 | Netherlands | 0.077 |
| Poland | 78.5 | Greece | 0.059 |
| Turkey | 78.3 | Poland | 0.047 |
| Slovakia | 77.4 | Italy | 0.040 |
| Hungary | 76.7 | Portugal | 0.040 |
| Malaysia | 76.1 | Japan | 0.012 |
| Lithuania | 75.7 | South Korea | 0.004 |

Table 15: Countries ranked by mean temperature.

| **Country** | **Mean Temperature (℃)** |
| --- | --- |
| Malaysia | 25.40 |
| Australia | 21.65 |
| Greece | 15.40 |
| Portugal | 15.15 |
| Italy | 13.45 |
| Spain | 13.30 |
| South Korea | 11.50 |
| Japan | 11.15 |
| Turkey | 11.10 |
| France | 10.70 |
| New Zealand | 10.55 |
| Hungary | 9.75 |
| Belgium | 9.55 |
| Ireland | 9.30 |
| Netherlands | 9.25 |
| Slovenia | 8.90 |
| United States | 8.55 |
| Germany | 8.50 |
| United Kingdom | 8.45 |
| Poland | 7.85 |
| Czech Republic | 7.55 |
| Denmark | 7.50 |
| Slovakia | 6.80 |
| Austria | 6.35 |
| Lithuania | 6.20 |
| Switzerland | 5.50 |
| Sweden | 2.10 |
| Finland | 1.70 |
| Norway | 1.50 |
| Canada | -5.35 |

Table 16: Countries ranked by % of adult population which is obese and % of adult population with diabetes.

| **Country** | **% obese** | **Country** | **% with diabetes** |
| --- | --- | --- | --- |
| United States | 36.2 | Malaysia | 16.7 |
| Turkey | 32.1 | Turkey | 11.1 |
| New Zealand | 30.8 | United States | 10.8 |
| Canada | 29.4 | Germany | 10.4 |
| Australia | 29.0 | Portugal | 9.8 |
| United Kingdom | 27.8 | Denmark | 8.3 |
| Hungary | 26.4 | Canada | 7.6 |
| Lithuania | 26.3 | Czech Republic | 7.0 |
| Czech Republic | 26.0 | Hungary | 6.9 |
| Ireland | 25.3 | South Korea | 6.9 |
| Greece | 24.9 | Spain | 6.9 |
| Spain | 23.8 | Austria | 6.6 |
| Norway | 23.1 | Slovakia | 6.5 |
| Poland | 23.1 | New Zealand | 6.2 |
| Germany | 22.3 | Poland | 6.1 |
| Finland | 22.2 | Slovenia | 5.9 |
| Belgium | 22.1 | Switzerland | 5.7 |
| France | 21.6 | Australia | 5.6 |
| Portugal | 20.8 | Finland | 5.6 |
| Sweden | 20.6 | Japan | 5.6 |
| Slovakia | 20.5 | Netherlands | 5.4 |
| Netherlands | 20.4 | Norway | 5.3 |
| Slovenia | 20.2 | Italy | 5.0 |
| Austria | 20.1 | France | 4.8 |
| Italy | 19.9 | Sweden | 4.8 |
| Denmark | 19.7 | Greece | 4.7 |
| Switzerland | 19.5 | Belgium | 4.6 |
| Malaysia | 15.6 | United Kingdom | 3.9 |
| South Korea | 4.7 | Lithuania | 3.8 |
| Japan | 4.3 | Ireland | 3.2 |

Table 17: Countries ranked by % of adult population with hypertension and % of adult population which smokes.

| **Country** | **% with hypertension** | **Country** | **% smoking** |
| --- | --- | --- | --- |
| Slovenia | 30.5 | Greece | 42.65 |
| Hungary | 30.0 | Austria | 35.15 |
| Lithuania | 29.3 | Czech Republic | 33.20 |
| Poland | 28.7 | Germany | 30.35 |
| Slovakia | 28.5 | Lithuania | 30.15 |
| Czech Republic | 27.9 | Spain | 29.20 |
| Portugal | 24.4 | Slovakia | 28.65 |
| Malaysia | 22.9 | Hungary | 28.40 |
| France | 22.0 | Poland | 28.05 |
| Italy | 21.2 | France | 27.70 |
| Austria | 21.0 | South Korea | 27.00 |
| Denmark | 20.6 | Turkey | 25.95 |
| Turkey | 20.3 | Netherlands | 25.05 |
| Germany | 19.9 | Italy | 24.00 |
| Ireland | 19.7 | Switzerland | 23.30 |
| Norway | 19.7 | Belgium | 23.25 |
| Finland | 19.4 | Portugal | 22.60 |
| Sweden | 19.3 | Norway | 22.25 |
| Spain | 19.2 | Malaysia | 22.20 |
| Greece | 19.1 | Ireland | 22.15 |
| Netherlands | 18.7 | Japan | 22.15 |
| Switzerland | 18.0 | Finland | 20.85 |
| Japan | 17.6 | Sweden | 20.60 |
| Belgium | 17.5 | Slovenia | 20.20 |
| New Zealand | 16.2 | United Kingdom | 19.15 |
| Australia | 15.2 | United States | 17.25 |
| United Kingdom | 15.2 | Denmark | 17.00 |
| Canada | 13.2 | Canada | 14.95 |
| United States | 12.9 | Australia | 14.90 |
| South Korea | 11.0 | New Zealand | 13.20 |

Table 18: Countries ranked by numbers of COVID-19 deaths and period from first case to date when death totals were retrieved.

| **Country** | **No. of COVID deaths** | **Country** | **Recording period (days)** |
| --- | --- | --- | --- |
| United States | 144,469 | Japan | 194 |
| United Kingdom | 45,639 | South Korea | 189 |
| Italy | 35,092 | United States | 188 |
| France | 30,185 | France | 184 |
| Spain | 28,429 | Australia | 183 |
| Belgium | 9,812 | Malaysia | 183 |
| Germany | 9,117 | Canada | 182 |
| Canada | 8,919 | Germany | 180 |
| Netherlands | 6,158 | Finland | 179 |
| Sweden | 5,697 | Italy | 179 |
| Turkey | 5,563 | Sweden | 177 |
| Switzerland | 1,977 | United Kingdom | 176 |
| Ireland | 1,763 | Spain | 175 |
| Portugal | 1,705 | Belgium | 173 |
| Poland | 1,655 | Switzerland | 153 |
| Japan | 994 | Austria | 151 |
| Austria | 711 | Norway | 151 |
| Denmark | 613 | Denmark | 150 |
| Hungary | 596 | Greece | 150 |
| Czech Republic | 365 | Lithuania | 149 |
| Finland | 329 | Netherlands | 149 |
| South Korea | 298 | New Zealand | 149 |
| Norway | 255 | Czech Republic | 147 |
| Greece | 201 | Ireland | 147 |
| Australia | 140 | Portugal | 146 |
| Malaysia | 123 | Poland | 144 |
| Slovenia | 115 | Slovenia | 144 |
| Lithuania | 80 | Hungary | 143 |
| Slovakia | 28 | Slovakia | 142 |
| New Zealand | 22 | Turkey | 137 |

Table 19: Countries ranked by death rate (deaths per million population) and time-adjusted death rate (deaths per million population per year) / log time-adjusted death rate.

| **Country** | **Deaths per million population** | **Country** | **Deaths per million population per year** | **Log deaths per million population per year** |
| --- | --- | --- | --- | --- |
| Belgium | 850.3 | Belgium | 1795.2 | 7.49 |
| United Kingdom | 675.8 | United Kingdom | 1402.5 | 7.25 |
| Spain | 608.3 | Spain | 1269.5 | 7.15 |
| Italy | 579.6 | Italy | 1182.5 | 7.08 |
| Sweden | 567.6 | Sweden | 1171.3 | 7.07 |
| France | 463.5 | France | 920.0 | 6.82 |
| United States | 439.0 | Ireland | 897.2 | 6.80 |
| Ireland | 361.1 | Netherlands | 882.9 | 6.78 |
| Netherlands | 360.2 | United States | 852.9 | 6.75 |
| Canada | 238.4 | Switzerland | 549.3 | 6.31 |
| Switzerland | 230.1 | Canada | 478.4 | 6.17 |
| Portugal | 166.7 | Portugal | 417.1 | 6.03 |
| Germany | 109.2 | Denmark | 258.6 | 5.56 |
| Denmark | 106.2 | Germany | 221.5 | 5.40 |
| Austria | 79.4 | Austria | 192.0 | 5.26 |
| Turkey | 66.7 | Turkey | 177.8 | 5.18 |
| Hungary | 61.5 | Hungary | 157.2 | 5.06 |
| Finland | 59.5 | Slovenia | 140.3 | 4.94 |
| Slovenia | 55.3 | Finland | 121.3 | 4.80 |
| Norway | 47.4 | Norway | 114.7 | 4.74 |
| Poland | 43.7 | Poland | 110.8 | 4.71 |
| Czech Republic | 34.1 | Czech Republic | 84.8 | 4.44 |
| Lithuania | 29.0 | Lithuania | 71.1 | 4.26 |
| Greece | 19.2 | Greece | 46.7 | 3.84 |
| Japan | 7.8 | Japan | 14.8 | 2.69 |
| South Korea | 5.8 | Slovakia | 13.2 | 2.58 |
| Australia | 5.6 | New Zealand | 11.3 | 2.42 |
| Slovakia | 5.1 | South Korea | 11.2 | 2.42 |
| New Zealand | 4.6 | Australia | 11.1 | 2.41 |
| Malaysia | 3.8 | Malaysia | 7.7 | 2.04 |

Table 20: Countries ranked by date of first COVID-19 case.

| **Country** | **Date of First Case** |
| --- | --- |
| Japan | 14 January 2020 |
| South Korea | 19 January 2020 |
| United States | 20 January 2020 |
| France | 24 January 2020 |
| Australia | 25 January 2020 |
| Malaysia | 25 January 2020 |
| Canada | 26 January 2020 |
| Germany | 28 January 2020 |
| Finland | 29 January 2020 |
| Italy | 29 January 2020 |
| Sweden | 31 January 2020 |
| United Kingdom | 01 February 2020 |
| Spain | 02 February 2020 |
| Belgium | 04 February 2020 |
| Switzerland | 24 February 2020 |
| Austria | 26 February 2020 |
| Norway | 26 February 2020 |
| Denmark | 27 February 2020 |
| Greece | 27 February 2020 |
| Lithuania | 28 February 2020 |
| Netherlands | 28 February 2020 |
| New Zealand | 28 February 2020 |
| Czech Republic | 01 March 2020 |
| Ireland | 01 March 2020 |
| Portugal | 02 March 2020 |
| Poland | 04 March 2020 |
| Slovenia | 04 March 2020 |
| Hungary | 05 March 2020 |
| Slovakia | 06 March 2020 |
| Turkey | 11 March 2020 |

Table 21: Countries ranked by date national measures were introduced and delay between first case and the introduction of national measures.

| **Country** | **National measures date** | **Country** | **National measures delay (days)** |
| --- | --- | --- | --- |
| Italy | 09 March 2020 | South Korea | 189 |
| Norway | 12 March 2020 | Sweden | 177 |
| Poland | 12 March 2020 | Japan | 93 |
| Denmark | 13 March 2020 | United States | 78 |
| Greece | 13 March 2020 | Australia | 65 |
| Netherlands | 15 March 2020 | Canada | 56 |
| Austria | 16 March 2020 | Spain | 55 |
| Czech Republic | 16 March 2020 | Germany | 54 |
| Finland | 16 March 2020 | France | 53 |
| Hungary | 16 March 2020 | Malaysia | 53 |
| Lithuania | 16 March 2020 | United Kingdom | 51 |
| Slovakia | 16 March 2020 | Finland | 47 |
| Slovenia | 16 March 2020 | Belgium | 42 |
| Switzerland | 16 March 2020 | Italy | 40 |
| Turkey | 16 March 2020 | Ireland | 26 |
| Belgium | 17 March 2020 | New Zealand | 26 |
| France | 17 March 2020 | Switzerland | 21 |
| Malaysia | 18 March 2020 | Austria | 19 |
| Portugal | 20 March 2020 | Portugal | 18 |
| Canada | 22 March 2020 | Lithuania | 17 |
| Germany | 22 March 2020 | Netherlands | 16 |
| United Kingdom | 23 March 2020 | Czech Republic | 15 |
| New Zealand | 25 March 2020 | Denmark | 15 |
| Ireland | 27 March 2020 | Greece | 15 |
| Spain | 28 March 2020 | Norway | 15 |
| Australia | 30 March 2020 | Slovenia | 12 |
| United States | 07 April 2020 | Hungary | 11 |
| Japan | 16 April 2020 | Slovakia | 10 |
| South Korea | 26 July 2020 | Poland | 8 |
| Sweden | 26 July 2020 | Turkey | 5 |

# Comparing the outcome variable with the model residuals

The outcome variable used in the models was log death rate, standardized to have mean 0 and standard deviation 1. In Table 22, countries are shown ranked by (a) the outcome variable, (b) residuals from Model 1 and (c) residuals from Model 2. The values of the outcome variable and the model residuals are also shown.

Table 22: Countries ranked by (a) the outcome variable, (b) residuals from Model 1 and (c) residuals from Model 2. The value of the outcome variable and the model residuals are shown.

| **Rank** | **(a) Outcome variable** | | **(b) Residuals from Model 1** | | **(c) Residuals from Model 2** | |
| --- | --- | --- | --- | --- | --- | --- |
| 1 | Belgium | 1.38 | Spain | 1.19 | Spain | 1.28 |
| 2 | United Kingdom | 1.23 | Italy | 0.85 | Sweden | 1.06 |
| 3 | Spain | 1.18 | Portugal | 0.59 | Italy | 0.86 |
| 4 | Italy | 1.13 | Belgium | 0.55 | France | 0.64 |
| 5 | Sweden | 1.13 | Ireland | 0.53 | Portugal | 0.57 |
| 6 | France | 0.99 | France | 0.53 | Ireland | 0.52 |
| 7 | Ireland | 0.97 | Malaysia | 0.50 | Belgium | 0.51 |
| 8 | Netherlands | 0.96 | Austria | 0.49 | United Kingdom | 0.45 |
| 9 | United States | 0.94 | United Kingdom | 0.46 | Malaysia | 0.44 |
| 10 | Switzerland | 0.68 | Switzerland | 0.37 | United States | 0.34 |
| 11 | Canada | 0.60 | Sweden | 0.36 | Austria | 0.28 |
| 12 | Portugal | 0.52 | Denmark | 0.32 | Canada | 0.19 |
| 13 | Denmark | 0.24 | Canada | 0.32 | Lithuania | 0.19 |
| 14 | Germany | 0.15 | Greece | 0.22 | Switzerland | 0.17 |
| 15 | Austria | 0.06 | Lithuania | 0.19 | Hungary | 0.16 |
| 16 | Turkey | 0.02 | Poland | 0.12 | Poland | 0.12 |
| 17 | Hungary | -0.05 | United States | 0.12 | Denmark | 0.09 |
| 18 | Slovenia | -0.12 | Hungary | 0.05 | South Korea | 0.01 |
| 19 | Finland | -0.21 | Finland | -0.09 | Greece | -0.04 |
| 20 | Norway | -0.24 | Turkey | -0.10 | Turkey | -0.17 |
| 21 | Poland | -0.26 | Netherlands | -0.21 | Finland | -0.23 |
| 22 | Czech Republic | -0.42 | South Korea | -0.24 | Slovenia | -0.29 |
| 23 | Lithuania | -0.52 | Slovenia | -0.41 | Netherlands | -0.32 |
| 24 | Greece | -0.77 | Norway | -0.50 | Slovakia | -0.61 |
| 25 | Japan | -1.45 | Slovakia | -0.51 | Norway | -0.68 |
| 26 | Slovakia | -1.51 | Germany | -0.88 | Germany | -0.73 |
| 27 | New Zealand | -1.60 | Japan | -1.02 | Czech Republic | -0.99 |
| 28 | South Korea | -1.61 | Czech Republic | -1.14 | Australia | -1.05 |
| 29 | Australia | -1.61 | Australia | -1.16 | Japan | -1.15 |
| 30 | Malaysia | -1.83 | New Zealand | -1.49 | New Zealand | -1.62 |

The outcome variable is standardized log death rate (deaths per million population per year).

# Data sources from which national measures dates were derived

A country was considered to have introduced national COVID-19 control measures once compulsory restrictions were adopted which applied to the whole country and included at least two of the following:

1. A limit on public gatherings to 30 people or fewer.
2. The closure of public buildings including non-essential shops, restaurants, bars, theatres and cinemas.
3. The closure of schools.
4. A requirement on the public to stay at home except for medical reasons, exercise, essential shopping and work that can’t be done remotely.
5. A requirement on the public to observe social distancing.

Details are given below of the sources used to determine the dates that national COVID-19 control measures were introduced in each country. Sources were accessed on 11^th^ of August 2020.

Australia

National measures date: 30 March 2020

Sources:

<https://en.wikipedia.org/wiki/COVID-19_pandemic_in_Australia>

<https://www.pm.gov.au/media/national-cabinet-statement>

“On 29 March, the Cabinet agreed to stricter limits to apply from midnight on the 30th: a limit on both indoor and outdoor gatherings of two people except weddings (5) funerals (10) and people of the same household or family; strong guidance to all Australians is to stay home unless for necessary shopping, health care, exercise, and work and study that can't be done remotely; public playgrounds, skate parks and outside gyms to be closed. It was left to individual states to enforce these guidelines. They also agreed to a moratorium on evictions for six months for both commercial and residential tenancies suffering financial distress.”

Austria

National measures date: 16 March 2020

Sources:

<https://en.wikipedia.org/wiki/COVID-19_pandemic_in_Austria>

<https://orf.at/stories/3158055/>

“On 16 March, a nationwide curfew went into force. Homes may only be left for a handful of specified reasons, see above. Non-essential work that cannot be done from home was stopped.”

Belgium

National measures date: 17 March 2020

Sources:

<https://en.wikipedia.org/wiki/COVID-19_pandemic_in_Belgium>

<https://www.belgium.be/en/news/2020/coronavirus_reinforced_measures>

“On 17 March, the National Security Council decided to take additional measures, based on the spread of COVID-19 in Belgium and on recommendations of experts. Stricter social distancing measures were imposed from noon the following day until 5 April, with non-essential travel prohibited, non-essential shops to close, gatherings banned, with penalties for corporate and individual persons who failed to comply with the restrictions.”

Canada

National measures date: 22 March 2020

Sources:

<https://en.wikipedia.org/wiki/COVID-19_pandemic_in_Canada#Provincial_and_territorial>

From a study of the COVID-19 control measures imposed by individual states, the conclusion was drawn that the conditions for “COVID-19 control measures”, as defined above, were met nationally from March the 22^nd^ 2020, this being the date that a state of emergency was declared in Nova Scotia.

Czech Republic

National measures date: 16 March 2020

Sources:

<https://en.wikipedia.org/wiki/COVID-19_pandemic_in_the_Czech_Republic>

“16 March – Starting at midnight, an hour after the nationwide quarantine declaration was approved the previous day, nearly 11 million Czech residents were placed under quarantine (see policies section below).[2] The Czech Republic became one of the first[39] countries in the EU to completely close its borders (with exemptions including international freight transport, see policies section below).[2] First three people were reported recovered.”

Denmark

National measures date: 13 March 2020

Sources:

<https://en.wikipedia.org/wiki/COVID-19_pandemic_in_Denmark>

<https://nyheder.tv2.dk/samfund/2020-03-11-danmark-lukker-ned-her-er-regeringens-nye-tiltag>

“Starting on 13 March 2020, all people working in non-essential functions in the public sector were ordered to stay home for two weeks.”

Finland

National measures date: 16 March 2020

Sources:

<https://en.wikipedia.org/wiki/COVID-19_pandemic_in_Finland>

“On 16 March, the Finnish Government, in cooperation with the President of Finland, declared a state of emergency in the country. A list of measures intended to slow down the spreading of the virus and to protect at-risk groups were implemented in accordance with the Emergency Powers Act (1552/2011), the Communicable Diseases Act (1227/2016), and other legislation.”

France

National measures date: 17 March 2020

Sources:

<https://en.wikipedia.org/wiki/COVID-19_pandemic_in_France>

<https://www.marianne.net/politique/emmanuel-macron-annonce-l-interdiction-des-deplacements-non-essentiels-des-mardi-midi>

“On 16 March (one day after the first round of the municipal elections), Emmanuel Macron announced the beginning of a lockdown period from the 17 March at noon.”

Germany

National measures date: 22 March 2020

Sources:

<https://en.wikipedia.org/wiki/COVID-19_pandemic_in_Germany>

<https://www.welt.de/politik/deutschland/article206725829/Coronavirus-Deutschland-Kontaktverbote-zu-mehr-als-zwei-Personen-Friseure-zu.html>

“On 22 March, the government and the federal states agreed for at least two weeks to forbid gatherings of more than two people and require a minimum distance of 1.5 metres (4 ft 11 in) between people in public except for families, partners or people living in the same household. Restaurants and services like hairdressers were to be closed.”

Greece

National measures date: 13 March 2020

Sources:

<https://en.wikipedia.org/wiki/COVID-19_pandemic_in_Greece>

<https://www.ekathimerini.com/250618/article/ekathimerini/news/malls-cafes-bars-eateries-to-close-as-coronavirus-cases-rise-to-190>

“On 13 March, the nationwide closure of all shopping centres, cafes, restaurants, bars, museums and archaeological sites and food outlets, excluding supermarkets, pharmacies and food outlets that offer take-away and delivery only, was announced.[398] On 14 March, all organised beaches and ski resorts were closed.”

Hungary

National measures date: 16 March 2020

Sources:

<https://en.wikipedia.org/wiki/COVID-19_pandemic_in_Hungary>

“Elementary and high schools were initially excluded from closure, due to an initial assessment that COVID-19 did not have as serious of an impact to children.[71][72][73] The Ministry of Human Capacities recommended that schools suspend field trips, open-air classes, and exchange programs.[74] On 13 March during a radio interview, Prime Minister Viktor Orbán said kindergarten was also excluded since parents would have to guarantee children's supervision, and teachers would be required to take unpaid leave.[75] That evening, Orbán announced that elementary and high schools would be closed to in-person classes effective 16 March.[76]

On 16 March, Prime Minister Orbán announced further restrictions, including ordering the cancellation of all events, and banning restaurants and cafes from operating beyond 3 p.m. Only grocery stores and pharmacies would be allowed to remain open past this time. In addition, it was announced that the country would allow entry to Hungarian citizens only.[77][77] In spite of the notices issued by operational staff about responsible behaviour and moderation, a 30-year-old security guard shared fake news on YouTube regarding the pandemic. He was the subject of police action.[78]”

Ireland

National measures date: 27 March 2020

Sources:

<https://en.wikipedia.org/wiki/COVID-19_pandemic_in_the_Republic_of_Ireland>

<https://www.gov.ie/en/publication/cf9b0d-new-public-health-measures-effective-now-to-prevent-further-spread-o/?referrer=/en/publication/539d23-stay-at-home-the-latest-public-health-measures-to-prevent-the-spread/>

“On 27 March, 302 new cases as well as 3 new deaths brought the total number of confirmed cases and deaths to 2,121 and 22, respectively.[52] Among the deaths was the country's first healthcare fatality, who was based in the east.[53] Taoiseach Leo Varadkar announced a series of measures which he summed up as: "Stay at Home" (subject to certain exemptions).”

Italy

National measures date: 09 March 2020

Sources:

<https://en.wikipedia.org/wiki/COVID-19_pandemic_in_Italy>

<https://edition.cnn.com/2020/03/09/europe/coronavirus-italy-lockdown-intl/index.html>

“On 9 March, the government announced that all sporting events in Italy would be cancelled until at least 3 April, but the ban does not include Italian clubs or national teams participating in international competitions.[242] In the evening, Conte announced in a press conference that all measures previously applied only in the so-called "red zones" had been extended to the whole country, putting approximately 60 million people in lockdown. Conte later proceeded to officially sign the new executive decree.[117][243]”

Japan

National measures date: 16 April 2020

Sources:

<https://en.wikipedia.org/wiki/COVID-19_pandemic_in_Japan>

“On 16 April, Abe expanded the state of emergency declaration to include every prefecture within the country.[11] Later on 4 May, Abe said that Japanese Cabinet would expand the state of emergency declaration until end of May.[73] Then on 14 May, Abe and his cabinet declared that Japanese Government decided to relieve the state of emergency declaration, excluding 8 prefectures like Tokyo, Kyoto Prefecture.[74] Some media expressed doubts about why only some of the easing standards were released under the name of comprehensive judgment.”

Lithuania

National measures date: 16 March 2020

Sources:

<https://en.wikipedia.org/wiki/COVID-19_pandemic_in_Lithuania>

“16 March: Two new cases were confirmed. Both patients were in Vilnius, and they had returned from Spain (Barcelona via Paris) and Germany.[30] Later in the day, three more cases were confirmed: one person in Telšiai who returned from the Dominican Republic on 8 March, and two people in Vilnius who returned from Austria.[31] Also on the same day, Lithuania was put under quarantine.”

Malaysia

National measures date: 18 March 2020

Sources:

<https://en.wikipedia.org/wiki/COVID-19_pandemic_in_Malaysia>

“Prime Minister Muhyiddin Yassin announced that Malaysia would enter into lockdown on 18 March 2020.”

Netherlands

National measures date: 15 March 2020

Sources:

<https://en.wikipedia.org/wiki/COVID-19_pandemic_in_the_Netherlands>

<https://nos.nl/artikel/2327194-alle-scholen-cafes-en-restaurants-tot-en-met-6-april-dicht-om-coronavirus.html>

“15 March: the total number of cases was 1,135. The Public Health Service (GGD) estimated that as of this day, 6,000 people in the Netherlands had been infected. This is because since 12 March people with mild complaints had not been tested any more.[38] Public measures were also tightened up. Schools and childcare centres will remain closed until 6 April, as well as cafés, restaurants, sports clubs, saunas, sex clubs and coffeeshops.”

New Zealand

National measures date: 25 March 2020

Sources:

<https://en.wikipedia.org/wiki/COVID-19_pandemic_in_New_Zealand>

“Beginning on 25 March, the Alert Level was moved to Level 4, putting the country into a nationwide lockdown.”

Norway

National measures date: 12 March 2020

Sources:

<https://en.wikipedia.org/wiki/COVID-19_pandemic_in_Norway>

<https://www.nrk.no/korona/status/>

“The Norwegian Directorate of Health introduced a number of measures from Thursday 12 March 2020.

- All educational institutions were closed and organized sports activities were to be discontinued.
- A number of events and businesses were closed, including cultural events, sports events, gyms and swimming pools. All establishments in the hospitality industry such as bars, pubs and clubs other than those serving food were to close, and any establishment serving food would have to ensure that visitors could stay at least 1 meter apart.
- Healthcare professionals working with patient care were prohibited from traveling abroad until 20 April 2020. The ban applied to both business travel and private travel.
- Everyone who had returned from trips outside Sweden and Finland since 27 February were to quarantine, regardless of whether they showed symptoms or not.
- Leisure travel was strongly discouraged. The Directorate discouraged travelling to work unless strictly necessary and encouraged avoiding public transport if possible, as well as avoiding crowded places.
- People were requested not to visit others in institutions with vulnerable groups (the elderly, psychiatry, prison etc.) and generally encouraged to limiting close contact with others.
- The public transport schedule was to run as normal, to ensure that people with critical social functions could get to and from work and be able to distance themselves from each other.”

Poland

National measures date: 12 March 2020

Sources:

<https://en.wikipedia.org/wiki/COVID-19_pandemic_in_Poland>

"Polish authorities' initial COVID-19 limitation strategy of laboratory testing, contact tracing, quarantining and monitoring intensified in mid-March with "lockdown" type measures. On 10 March, authorities cancelled all mass events, defined as those allowing 1000 or more participants in the case of stadiums or other events outside of buildings, and those allowing 500 or more participants in the case of events in buildings.[11] Cultural institutions, such as philharmonic orchestras, operas, theatres, museums, and cinemas, had their activities suspended beginning on 12 March 2020.[12]

All schools in Poland were closed starting on 12 March, with a reopening initially scheduled for 25 March 2020.[13] The closure was extended to 10 April, with schools being required to carry out online classes with their students. As of 20 March, the dates of final exams for eighth (final) year of primary school and matura, the exam during the final year of secondary school (liceum or technikum), remained unchanged.[56]"

Portugal

National measures date: 20 March 2020

Sources:

<https://www.gov.uk/government/news/portugal-coronavirus-covid-19-state-of-emergency#:~:text=On%2018%20March%202020%20the,virus%20(COVID%2D19).>

“On 18 March 2020 the President of Portugal, Marcelo Rebelo de Sousa, announced a State of Emergency to be brought in by the Portuguese Government. The State of Emergency legislation published on 20 March brings into force a series of measures to limit the spread of the virus (COVID-19). Cases of coronavirus (COVID-19) have been confirmed in Portugal.”

Slovakia

National measures date: 16 March 2020

Sources:

<https://en.wikipedia.org/wiki/COVID-19_pandemic_in_Slovakia>

“12 March 2020 - Emergency declared,[9] all event venues closed[10]

13 March 2020 - Compulsory 14-day quarantine upon returning from abroad,[11] restarting border controls, international passenger transportation halted[citation needed]

15 March 2020 - Emergency declared in health care,[12] face masks compulsory in public transport and shops[13]

16 March 2020 - Non-essential stores closed”

Slovenia

National measures date: 16 March 2020

Sources:

<https://en.wikipedia.org/wiki/COVID-19_pandemic_in_Slovenia>

“16 March 2020: The government closed all restaurants and bars. Public parking places in Ljubljana, Maribor, Murska Sobota were made free for the time being; all educational institutions, including kindergartens, primary and secondary schools, closed down. 34 new cases, 253 total out of 6,712 tests (until 14:20).[26] Infection with COVID-19 has been confirmed with one employee from nursing home in Štore and suspected with an employee from nursing home in Šmarje pri Jelšah; 12 more employees were ordered to self-isolate. 6 infections were confirmed among Red Cross volunteers taking body temperature of the travellers on the border crossings with Italy.”

South Korea

National measures date: 26 July 2020

Sources:

<https://en.wikipedia.org/wiki/COVID-19_pandemic_in_South_Korea>

No evidence was found that government measures ever met the conditions for “COVID-19 control measures”, as defined above.

Spain

National measures date: 28 March 2020

Sources:

<https://en.wikipedia.org/wiki/COVID-19_pandemic_in_Spain>

“On 28 March, the Spanish government banned all non-essential activity,[142][143] providing affected workers with paid recoverable leave unless they provide an essential service, work remotely, are on sick leave, or have their contracts suspended.”

Sweden

National measures date: 26 July 2020

Sources:

<https://en.wikipedia.org/wiki/COVID-19_pandemic_in_Sweden>

No evidence was found that government measures ever met the conditions for “COVID-19 control measures”, as defined above.

Switzerland

National measures date: 16 March 2020

Sources:

<https://en.wikipedia.org/wiki/COVID-19_pandemic_in_Switzerland>

“On 16 March, the Federal Council announced[40] further measures, and a revised ordinance.[41][42][43] Measures include the closure of bars, shops and other gathering places until 19 April, but leaves open certain essentials, such as grocery shops, pharmacies, (a reduced) public transport and the postal service.[44]”

Turkey

National measures date: 16 March 2020

Sources:

<https://en.wikipedia.org/wiki/COVID-19_pandemic_in_Turkey>

“On 15 March, the Ministry of Culture and Tourism announced that between 16 and 30 March all libraries in Turkey would be closed.[103] The Ministry of the Interior announced that pavilions, discotheques, bars and night clubs would be closed temporarily starting from 10:00 on 16 March.”

United Kingdom

National measures date: 23 March 2020

Sources:

<https://en.wikipedia.org/wiki/COVID-19_pandemic_in_the_United_Kingdom>

“Having previously advised the public to avoid pubs and restaurants, on 23 March, Boris Johnson announced in a television broadcast that measures to mitigate the virus were to be tightened to protect the NHS, with wide-ranging restrictions on freedom of movement, enforceable in law,[9] under a stay-at-home order which would last for at least three weeks.”

United States

National measures date: 07 April 2020

Sources:

<https://en.wikipedia.org/wiki/COVID-19_pandemic_in_the_United_States>

From a study of the COVID-19 control measures imposed by individual states tabulated here:

<https://en.wikipedia.org/wiki/U.S._state_and_local_government_responses_to_the_COVID-19_pandemic>

the conclusion was drawn that the conditions for “COVID-19 control measures”, as defined above, were met nationally from April the 7^th^ 2020, this being the date that a “stay at home” was ordered in South Carolina.
